# Prognostic Implications of Cardiac Magnetic Resonance Imaging Characteristics in Primary Mitral Regurgitation

**DOI:** 10.1016/j.jacadv.2025.101838

**Published:** 2025-06-25

**Authors:** Alexandre Altes, Vincent Hanet, David Vancraeynest, Agnès Pasquet, Achwaq Lebouazda, François Delelis, Hélène Dumortier, Valentina Silvestri, Manuel Toledano, Jean-Louis Vanoverschelde, Sylvestre Maréchaux, Bernhard L. Gerber

**Affiliations:** a Division of Cardiology, Department of Cardiovascular Diseases, Cliniques Universitaires St. Luc, Pôle de Recherche Cardiovasculaire (CARD), Institut de Recherche Expérimentale et Clinique (IREC), Université Catholique de Louvain, Brussels, Belgium; b GCS-Groupement des Hôpitaux de l’Institut Catholique de Lille/Lille Catholic Hospitals, Heart Valve Center, Cardiology Department, ETHICS EA 7446, DataCoeur, Lille Catholic University, Lille, France

**Keywords:** cardiac magnetic resonance imaging, left atrial function, mitral valve surgery, primary mitral regurgitation, prognosis

## Abstract

**Background:**

Knowledge remains limited regarding the relationship between cardiac magnetic resonance (CMR) preoperative characteristics and postoperative clinical outcomes in primary mitral regurgitation (MR).

**Objectives:**

The authors assessed the prognostic value of CMR preoperative characteristics in patients with primary MR due to prolapse or flail undergoing mitral valve surgery.

**Methods:**

We retrospectively studied 284 patients (median age 61 years, 24% women) with chronic significant primary MR, who underwent CMR and echocardiography (echo) prior to mitral valve repair surgery. The endpoint was a composite of all-cause mortality, hospitalization for heart failure, stroke, or life-threatening ventricular arrhythmia.

**Results:**

Over a median follow-up of 7.3 years (Q1-Q3: 3.4-10.5), adverse events occurred in 36 (13%) patients. CMR-left atrial emptying fraction (LAEF) (HR: 1.84 [95% CI: 1.32-2.56]; *P* < 0.001), CMR-right ventricular ejection fraction (HR: 1.36 [95% CI: 1.00-1.84]; *P* = 0.047), and CMR-indexed aortic forward stroke volume (HR: 1.40 [95% CI: 0.99-2]; *P* = 0.059) were each associated with a higher risk of adverse outcomes (HR for decrease in 1 SD). After adjusting for clinical and imaging risk factors, reduced CMR-LAEF remained independently associated with adverse prognosis (adjusted HR: 1.78 [95% CI: 1.27-2.48]; *P* < 0.001). Patients with CMR-LAEF <30% had higher 5-year event rates (28% vs 4%; *P* < 0.001) and were at a substantially higher risk of adverse outcomes (adjusted HR: 3.78 [95% CI: 1.83-7.80]; *P* < 0.001), with added prognostic value confirmed by multiple performance model metrics.

**Conclusions:**

In patients with primary MR, among CMR and echo preoperative characteristics, reduced CMR-LAEF, with a threshold value of 30%, is markedly associated with an increased risk of postoperative adverse outcomes.

Mitral valve (MV) surgery, preferably using repair techniques when MV anatomy is favorable, is the only effective treatment for achieving long-term favorable outcomes in patients with significant primary mitral regurgitation (MR).^1^ Current guidelines recommend MV surgery for patients exhibiting MR-related symptoms and/or left ventricular (LV) dysfunction or dilatation, based on MV repair feasibility and operative risk.^2^^,^^3^ However, beyond LV impairment, long-standing MR impacts global cardiac remodeling and hemodynamics.^4^^,^^5^ Consequently, some patients undergoing MV surgery may still experience adverse postoperative events due to irreversible damage to cardiac chambers or pulmonary vasculature. Notably, a strong association between the left atrial (LA) size or function and outcome has been reported in primary MR.^6^, ^7^, ^8^ Thus, greater granularity in clinical risk stratification for primary MR patients undergoing MV surgery is needed.

Cardiac magnetic resonance (CMR) imaging has become increasingly important in many primary MR patients for complete assessment of MR severity. Two pivotal studies have linked the CMR-based quantification of MR and progression to MV surgery and/or mortality.^9^^,^^10^ However, it remains unclear whether CMR, beyond clinical and echocardiographic assessment, can improve postoperative clinical risk stratification of primary MR patients. To date, few studies have investigated the relationship between CMR preoperative characteristics and postoperative outcome, focusing on surrogate endpoints such as postoperative LV remodeling or symptom changes.^11^^,^^12^ We therefore sought to assess the prognostic value of CMR preoperative characteristics in patients with significant primary MR undergoing MV surgery. Specifically, we hypothesized that the preoperative assessment of LA function by CMR would be associated with outcome, independently from clinical, echocardiographic, and other CMR characteristics.

## Methods

### Study sample

This study involved patients with primary chronic significant MR due to valve prolapse or flail referred for MV repair surgery in 2 heart valve centers (Brussels, Lille) between 2005 and 2022 who had undergone CMR and echo prior to scheduled MV repair surgery. The Brussels cohort was approved by IRB (NCT02974218, 2017 07MAR123 MV-3D Doppler, 2020-01AVR 192 Valve disease). The Lille cohort was part of the ongoing COHORTE-IM (NCT03962023), authorized by the French Ethics Committee (N° ID-RCB: 2019-A00869-48 Sud Méditérannée IV). Inclusion criteria were patients at least 18 years of age with moderate-to-severe and severe (according to American Society of Echocardiography/European Association of Cardiovascular Imaging guidelines) primary MR who were scheduled to undergo MV surgery.^2^^,^^3^ Exclusion criteria were acute MR, MR from another etiology than MV prolapse or flail, MV replacement, prior cardiac surgery, more than mild aortic stenosis, aortic regurgitation or mitral stenosis, prosthetic valve or intracardiac shunt, pregnancy, standard contraindication for CMR, poor echocardiographic image quality, missing CMR data (n = 13), off-axis LA imaging on 4- and 2-chamber views (n = 2), residual postoperative MR ≥ moderate (n = 10), refusal to participate in the study, and evidence of ischemic myocardial scar. Postoperative MR ≥ moderate was considered as an exclusion criterion because of its influence on postoperative clinical outcomes, irrespective of preoperative characteristics.

In each center, patients were prospectively invited to undergo CMR during hospitalization for the preoperative workup of primary MR. However, CMR was not systematically performed in all eligible patients, as its use varied over time in line with evolving clinical practice. CMR data were entered into an electronic database in each center and retrospectively pooled. Clinical data were obtained by chart review. The Logistic EuroSCORE II was calculated for all patients.^13^ Significant coronary artery disease (CAD) was considered >50% epicardial stenosis.

### Echocardiography

All patients had comprehensive Doppler-echocardiographic exams performed using commercially available ultrasound systems by experienced echocardiographers and analyzed locally in each center by the different investigators according to European Association of Cardiovascular Imaging/American Society of Echocardiography guidelines.^14^ The etiology of MR was preoperatively assessed by echocardiography and confirmed on surgical reports. MR severity was graded according to a multiparametric approach, as recommended by guidelines. MR effective regurgitant orifice area (EROA) and regurgitant volume (Echo-RegVol) values were computed by the Proximal Isovelocity Surface Area (PISA) method.

### Cardiac magnetic resonance imaging

Details regarding the CMR assessment are provided in the Supplemental Appendix.

**Figure 1:**
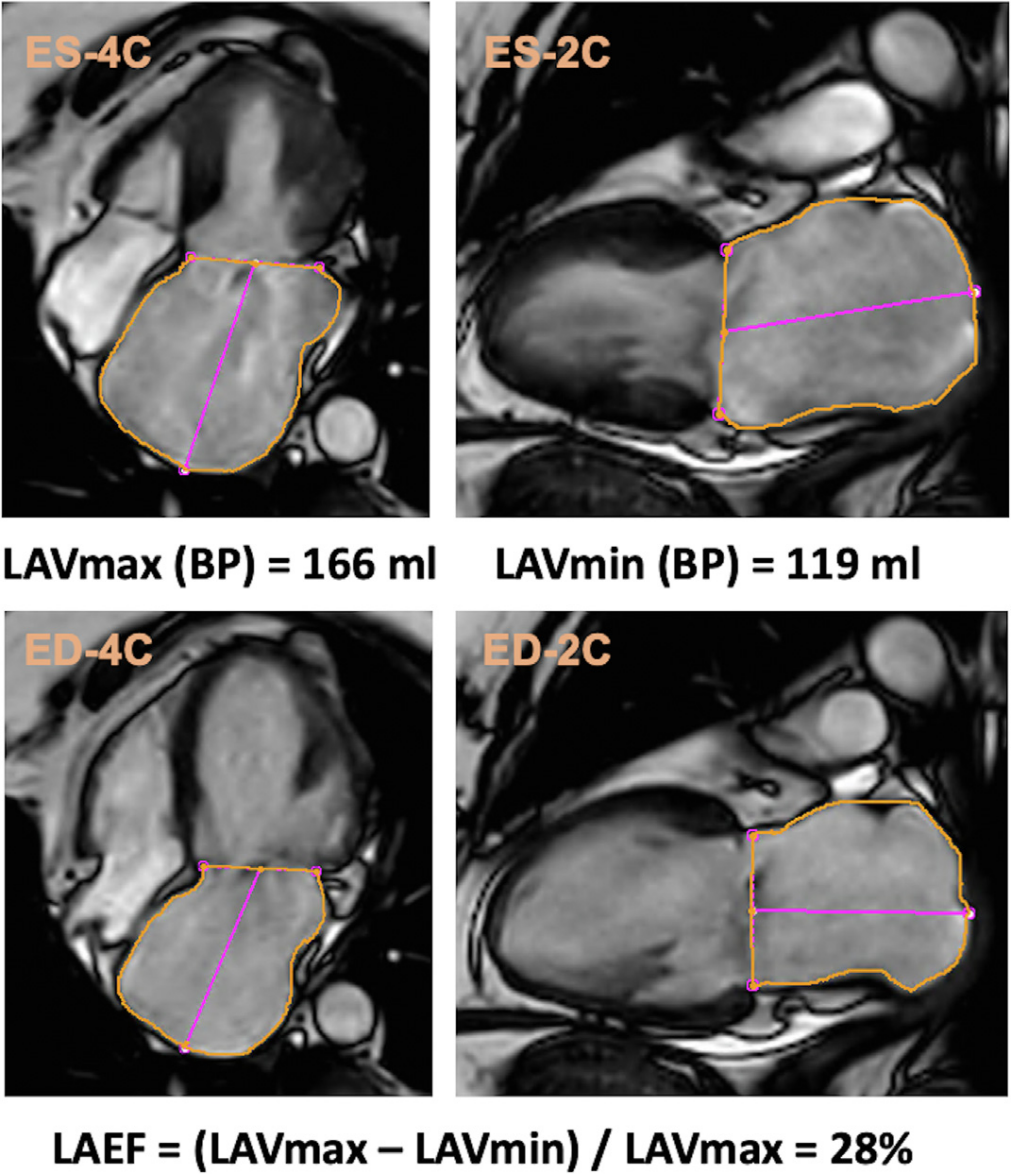
Measurement of Cardiac Magnetic Resonance-Left Atrial Emptying Fraction This figure displays the measurement and calculation of CMR-LAEF by the biplane (BP) area-length method (4- and 2-chamber view). 4C = 4-chamber view; 2C = 2-chamber view; CMR = cardiac magnetic resonance; ED = end-diastolic frame; ES = end-systolic frame; LAVmax = maximal (end-systolic) left atrial volume; LAVmin = minimal (end-diastolic) left atrial volume; LAEF = left atrial emptying fraction.

CMR studies were performed on clinical scanners and analyzed locally by experienced operators blinded to the echocardiographic data of the patient. Ventricular chamber assessment was performed in accordance with guidelines.^15^ Mitral regurgitant volume (CMR-RegVol) was calculated by subtracting the aortic stroke volume from the LV total stroke volume. Mitral regurgitant fraction (CMR-RegFrac) was defined as CMR-RegVol divided by the LV total stroke volume, expressed as a percentage. Late gadolinium enhancement (LGE) imaging was performed 10 to 15 minutes after intravenous administration of gadolinium contrast agent using inversion-recovery pulse sequences. LA maximal (CMR-LAVmax) and minimal (CMR-LAVmin) volumes were assessed using the biplane area-length method (2- and 4-chamber cine images) (Figure 1).^16^ LA emptying fraction (CMR-LAEF) was calculated as follows: (LAVmax−LAVmin)/LAVmax, expressed as a percentage.

### Clinical decision and follow-up

All patients underwent MV repair surgery within 6 months following baseline evaluation based on recommendation by the respective heart teams of each institution with the approval of the patient’s cardiologist in accordance with guidelines. The majority of patients were followed in our institution’s outpatient clinics. Others were followed in public hospitals or private practices by referring cardiologists working in coordination with our centers. Follow-up information was obtained retrospectively. Events were ascertained by chart review, direct patient interview, and physical examination and/or by repeated follow-up letters, questionnaires, and telephone calls to physicians, patients, and (if necessary) next of kin. Follow-up data were complete for 270 of the 284 patients (95.1%) at the time of analysis. The endpoint was a composite of all-cause mortality, hospitalization for heart failure, stroke, or life-threatening ventricular arrhythmia. Patients who underwent redo MV surgery were censored at the time of reintervention.

### Statistical analysis

Details regarding the statistical methods are provided in the Supplemental Appendix. Data were analyzed with R version 4.1.1 and GraphPad Prism. Quantitative data are reported as median (25th-75th percentile), and categorical variables as numbers and percentages. Time-to-event analyses were performed using Cox proportional hazards models, including a multivariable model built through stepwise backward selection based on the Akaike information criterion, starting from a full model that included all candidate covariates associated with outcome in univariable analyses.

A threshold for CMR-LAEF was identified using spline analysis, and patients were stratified by CMR-LAEF ≥30% or <30%. Survival analyses were conducted using Kaplan-Meier curves and Cox regression analyses. Model robustness was confirmed via bootstrapping and Poisson regression with a robust sandwich estimator. The incremental prognostic value of LAEF <30% was tested using likelihood ratio tests and multiple discrimination metrics, including net reclassification index, integrative discrimination index, and Harrell C-statistic with 95% bootstrap CIs. Factors associated with CMR-LAEF were analyzed using Pearson coefficient correlations and linear regression models. All analyses considered a 2-tailed *P* value of <0.05 as statistically significant.

## Results

### Baseline characteristics

Of the 284 patients included (median age 61 [Q1-Q3: 51-69] years), 24% were women (Table 1, Table 2). There were no significant differences in CMR-LAEF between sexes (women: 42% [32.5%-51%], men: 44% [38%-52%], *P* = 0.138). Women were older than men (66 [59-71] years vs 59 [50-67] years, *P* = 0.001). The median time between echo and CMR was 14 [Q1-Q3: 1-39] days.

**Table 1:** Demographic, Clinical, and Surgical Characteristics of the Study Sample

	All (N = 284)	No Event (n = 248)	Event (n = 36)	Overall *P* Value
Clinical characteristics				
Age (y)	61 (51-69)	59 (51-67)	69.5 (63-74)	**<0.001**
Women	69 (24%)	53 (21%)	16 (44%)	**0.005**
Body surface area (m^2^)	1.93 (1.79-2.05)	1.94 (1.81-2.06)	1.83 (1.60-1.99)	**0.004**
Body mass index (kg/m^2^)	24.7 (22.7-26.9)	24.6 (22.7-26.6)	25.4 (22.5-27.6)	0.621
NYHA functional class				0.129
I	118 (41.5%)	108 (43.5%)	10 (28%)	
II	108 (38%)	93 (37.5%)	15 (42%)	
III/IV	58 (20%)	47 (19%)	11 (31%)	
Hypertension	109 (38%)	96 (39%)	13 (36%)	0.907
Diabetes mellitus	7 (2%)	6 (2%)	1 (3%)	1.000
Dyslipidemia	84 (30%)	72 (29%)	12 (33%)	0.739
Coronary artery disease	40 (14%)	30 (12%)	10 (28%)	**0.023**
History of atrial fibrillation	47 (16.5%)	34 (14%)	13 (36%)	**0.002**
eGFR (ml/min/1.73 m^2^)	88 (66-110)	90 (69-112)	66 (52-89)	**<0.001**
Surgical characteristics				
EuroSCORE II (%)	0.99 (0.68-1.70)	0.96 (0.67-1.56)	1.55 (0.96-2.68)	**<0.001**
Surgical approach				0.211
Robotic-assisted surgery	69 (24%)	64 (26%)	5 (14%)	
Median sternotomy	193 (68%)	164 (66%)	29 (81%)	
Video-assisted minimally invasive surgery	22 (8%)	20 (8%)	2 (6%)	
Associated CABG	28 (10%)	19 (8%)	9 (25%)	**0.005**
Associated tricuspid annuloplasty	91 (32%)	78 (31.5%)	13 (36%)	0.712

Values are median (25th-75th percentile) or n (%). The **bold font** indicates statistical significance (*P* < 0.05).

CABG = coronary artery bypass graft; eGFR = estimated glomerular filtration rate.

**Table 2:** Echocardiographic and CMR Characteristics of the Study Sample

	All (N = 284)	No Event (n = 248)	Event (n = 36)	Overall *P* Value
Echocardiographic characteristics				
LVEDD (mm)	58 (55-62)	59 (55-62)	56 (52-59.5)	**0.028**
LVESD (mm)	35 (31-40)	36 (32-40)	33.5 (29-40)	0.233
Echo-LVEF (%)	64 (60-70)	64 (60-69)	66.5 (60.5-72)	0.208
Echo-indLAV (mL/m^2^)	54 (43-71)	54 (43-71)	49 (39-72)	0.514
TRmax PG (mm Hg) (n = 238)	26 (21-35)	26 (21-33)	29 (23-42.5)	0.104
MR-EROA (mm^2^) (n = 269)	51 (40-65)	51 (40-65)	45 (33-63)	0.111
Echo-RegVol (mL) (n = 269)	75 (62-91)	75 (62-91)	73 (56-84)	0.316
MR prolapse localization				0.513
Anterior	23 (8%)	20 (8%)	3 (8%)	
Posterior	201 (71%)	173 (70%)	28 (78%)	
Bi-leaflet	60 (21%)	55 (22%)	5 (14%)	
Flail leaflet	122 (43%)	109 (44%)	13 (36%)	0.479
CMR characteristics				
CMR-indLVEDV (mL/m^2^)	116 (99-129)	117 (101-130)	107 (88-122)	**0.016**
CMR-indLVESV (mL/m^2^)	42 (34-52)	42 (35-53)	35 (29-46)	**0.011**
CMR-indLV mass (g/m^2^)	74.5 (66-82.5)	75 (67-82.5)	72 (60-82)	0.187
CMR-LVEF (%)	63 (58-67)	63 (58-67)	64 (59-70)	0.256
CMR-ind aortic forward volume (mL/m^2^)	37 (30-42)	38 (31-42.5)	31 (25-40)	**0.006**
CMR-RegVol (mL)	62 (47-86)	62 (50-86)	52 (40-83)	0.051
CMR-RegFrac (%)	49 (38-57)	49 (38-57)	49 (38-58)	0.633
CMR-indRVEDV (mL/m^2^)	77 (66-90)	79 (67-90)	74 (62-89)	0.261
CMR-indRVESV (mL/m^2^)	38 (30-47)	38 (30-47)	38 (31-49)	0.633
CMR-RVEF (%)	51 (46-56)	52 (46-57)	49.5 (44-52)	**0.014**
LV fibrosis (n = 236)	51 (22%)	45 (22%)	6 (21%)	1.000
CMR-ind end-systolic LAV (mL/m^2^)	76 (61-98)	75.5 (60-97)	78 (63-116)	0.244
CMR-ind end-diastolic LAV (mL/m^2^)	42 (31-58)	42 (29.5-56)	49 (35-84)	**0.021**
CMR-LAEF (%)	44 (37-52)	45 (38.5-52)	38 (20.5-43)	**<0.001**

Values are median (25th-75th percentile) or n (%). The **bold font** indicates statistical significance (*P* < 0.05).

CMR = cardiac magnetic resonance; EDD = end-diastolic diameter; EDV = end-diastolic volume; EF = ejection fraction; ESD = end-systolic diameter; ESV = end-systolic volume; ind = indexed to body surface area; LAV = left atrial volume; LAEF = left atrial emptying fraction; LV = left ventricle/ventricular; MR = mitral regurgitation; MR-EROA = mitral regurgitant effective regurgitant orifice area; RegFrac = mitral regurgitant fraction; RegVol = mitral regurgitation volume; RV = right ventricle/ventricular; TRmax PG = tricuspid regurgitation peak pressure gradient.

### Relationship between clinical preoperative characteristics and postoperative adverse events

During a median follow-up period of 7.3 years (Q1-Q3: 3.4-10.5 years), adverse events occurred in 36 (13%) patients (all-cause mortality [n = 19], hospitalization for heart failure [n = 10], stroke [n = 5], or life-threatening ventricular arrhythmia [symptomatic sustained ventricular tachycardia, n = 2]). Nine patients underwent redo MV surgery during follow-up due to significant recurrent MR, and 1 additional patient was reoperated for endocarditis. Of the 9 patients with recurrent MR, 1 experienced the primary outcome (hospitalization for heart failure), while the remaining 8 did not. The median time between CMR and MV surgery was 26 (Q1-Q3: 2-58) days.

On univariable Cox analyses, age (HR per increase of 1 year 1.06 [95% CI: 1.03-1.10]; *P* < 0.001), female sex (HR: 2.21 [95% CI: 1.14-4.28]; *P* = 0.019), history of atrial fibrillation (AF) (HR: 2.82 [95% CI: 1.43-5.57]; *P* = 0.003), CAD (HR: 3 [95% CI: 1.44-6.25]; *P* = 0.003), estimated glomerular filtration rate (eGFR) (HR: 1.02 [95% CI: 1.01-1.04]; *P* = 0.001, for each 1 mL/min/1.73 m^2^ reduction in eGFR), and EuroSCORE II (HR per increase of 1%: 1.40 [95% CI: 1.18-1.67]; *P* = 0.001) were each associated with an increased risk of adverse events. A trend for higher risk of adverse outcome was found for NYHA functional class ≥3 (HR: 1.83 [95% CI: 0.89-3.74]; *P* = 0.099).

### Relationship between echo and CMR preoperative characteristics and postoperative adverse events

**Figure 2:**
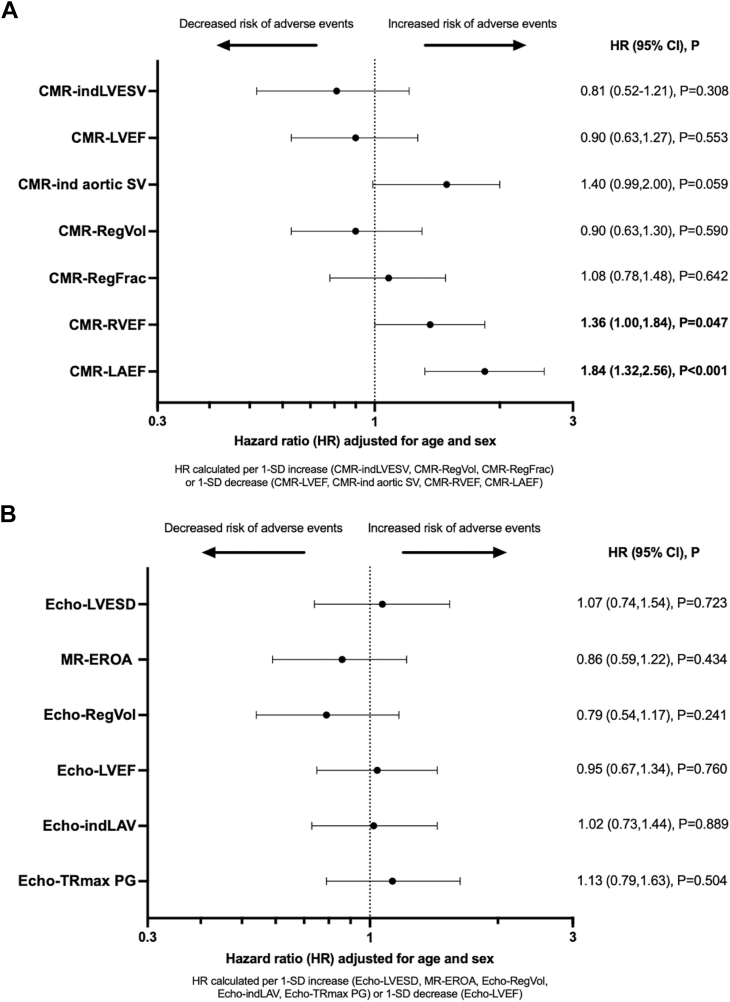
Age- and Sex-Adjusted Cox Survival Analysis for Imaging Characteristics These forest plots display the relationship between CMR (A) and echo (B) characteristics and the risk of adverse events following MV surgery. HRs are calculated for a 1-SD increase (CMR-indLVESV, CMR-RegVol, CMR-RegFrac, Echo-indLAV, Echo-LVESD, Echo-RegVol, Echo-TRmax PG, MR-EROA) or a 1-SD decrease (CMR-ind aortic SV, CMR-LVEF, CMR-RVEF, CMR-LAEF, Echo-LVEF). CMR = cardiac magnetic resonance; EF = ejection fraction; ESD = end-systolic diameter; ind = indexed to body surface area; LAEF = left atrial emptying fraction; LAV = left atrial volume; LV = left ventricular; MR-EROA = mitral regurgitant effective regurgitant orifice area; MV = mitral valve; RegFrac = regurgitant fraction; RegVol = regurgitant volume; RV = right ventricular; SV = stroke volume; TRmax PG = tricuspid regurgitation peak pressure gradient.

On age- and sex-adjusted Cox analyses, CMR-ind aortic forward stroke volume (adjusted HR per decrease of 1 SD: 1.40 [95% CI: 0.99-2]; *P* = 0.059), CMR-RVEF (adjusted HR per decrease of 1 SD: 1.36 [95% CI: 1.00-1.84]; *P* = 0.047), and CMR-LAEF (adjusted HR per decrease of 1 SD: 1.84 [95% CI: 1.32-2.56]; *P* < 0.001) were each associated with an increased risk of adverse events (Figure 2, Central Illustration, left panel). Conversely, no echo (MR-EROA, Echo-RegVol) or CMR (CMR-RegVol, CMR-RegFrac) MR quantitative parameters were associated with increased risk of postoperative clinical adverse events (all *P* > 0.241). In patients who underwent LGE imaging (n = 236), no relationship was found between the presence of LGE and outcome (adjusted HR: 0.89 [95% CI: 0.33-2.39]; *P* = 0.824).

**Central Illustration:**
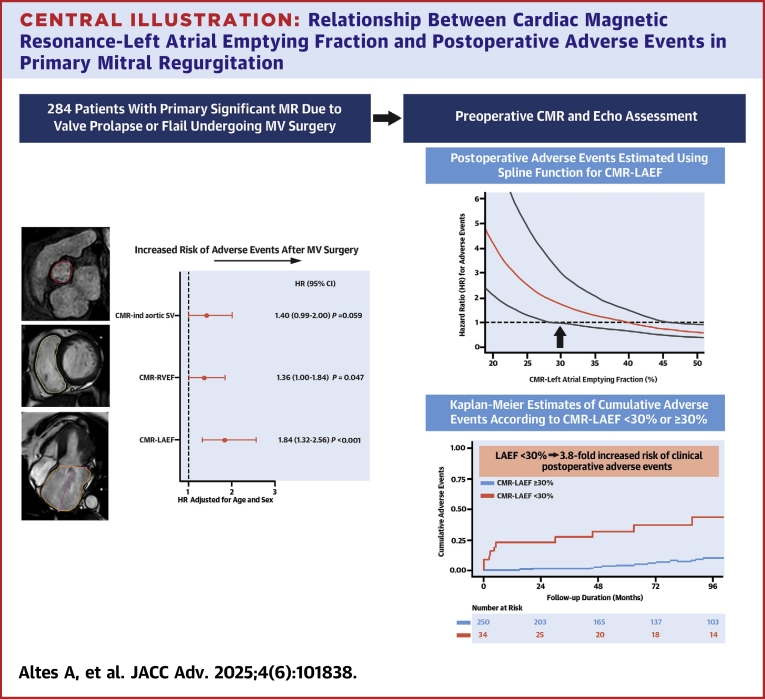
Relationship Between Cardiac Magnetic Resonance-Left Atrial Emptying Fraction and Postoperative Adverse Events in Primary Mitral Regurgitation The forest plot (left) shows the association of CMR-LAEF, CMR-indexed aortic SV, and CMR-RVEF with postoperative adverse events after mitral valve surgery, using age- and sex-adjusted Cox models. HRs are expressed per 1-SD decrease. The upper right panel presents a spline-based analysis of CMR-LAEF and the risk of postoperative adverse events. HRs (solid red line) and 95% CI (dashed lines) were estimated from a Cox model, with CMR-LAEF modeled as a penalized spline. Risk significantly increases below 30% (indicated by arrow). The lower right panel displays Kaplan-Meier estimates of cumulative adverse events stratified by CMR-LAEF < vs ≥30%. CMR = cardiac magnetic resonance; EF = ejection fraction; ind = indexed to body surface area; LAEF = left atrial emptying fraction; LAV = left atrial volume; LV = left ventricular; RV = right ventricular; SV = stroke volume.

A multivariable Cox model was built using stepwise backward selection based on the Akaike information criterion, starting from a full model including all candidate covariates (those associated with outcome in univariable analyses: age, female sex, history of AF, eGFR, NYHA functional class ≥3, EuroSCORE II, CAD, CMR-ind aortic forward stroke volume, CMR-RVEF, and CMR-LAEF). After backward selection, covariates retained in the final Cox multivariable model were age, female sex, history of AF, CAD, and CMR-LAEF. Using this parsimonious model, CMR-LAEF was independently associated with an increased risk of adverse events (adjusted HR: 1.78 [95% CI: 1.27-2.48]; *P* < 0.001) (Table 3).

**Table 3:** Relative Risk of Postoperative Adverse Events Associated With CMR-LAEF (as a Continuous Covariate)

Multivariable Analysis	HR (95% CI)	*P* Value
Age (per increase of 1 y)	1.03 (0.99-1.07)	0.089
Women	2.48 (1.18-5.22)	**0.017**
History of atrial fibrillation	2.14 (0.99-4.62)	0.052
Coronary artery disease	3.82 (1.72-8.47)	**<0.001**
CMR-LAEF (per decrease of 1 SD)	1.78 (1.27-2.48)	**<0.001**

**Bold** indicates statistical significance (*P* < 0.05).

Abbreviations as in Table 2.

### Relationship between CMR-LAEF and postoperative adverse events

We used spline curve graphs to analyze the relationship between CMR-LAEF and adverse events (Central Illustration, right upper panel). An optimal threshold value of 30% for CMR-LAEF was identified. Patients with CMR-LAEF <30% were older, more often women, presented more frequently with a history of AF, had higher EuroSCORE II, and were more likely to undergo associated coronary artery bypass graft (CABG) at the time of MV surgery (Supplemental Table 1) (all *P* < 0.026). Also, they had larger indexed LA volumes, higher CMR-RegFrac, higher estimated pulmonary pressures, lower CMR-LVEF, and indexed aortic forward stroke volume (Supplemental Table 2) (all *P* < 0.028).

Patients with CMR-LAEF <30% showed higher rates of adverse events at 5-year follow-up compared to those with CMR-LAEF ≥30% (28% vs 4%, *P* < 0.001) (Central Illustration, right lower panel). After adjusting for age, female sex, CAD, and history of AF, CMR-LAEF <30% remained associated with an increased risk of adverse events (adjusted HR: 3.78 [95% CI: 1.83-7.80]; *P* < 0.001) (Table 4). These results were confirmed by bootstrapping with 1,000 replicates and Poisson regression analysis (Supplemental Table 3). Substituting CAD with “associated CABG” yielded similar results (adjusted HR: 3.43 [95% CI: 1.65-7.12]; *P* < 0.001). Supplemental Table 4 shows the distribution of events and nonevents across 2 × 2 × 2 covariate combinations (sex, history of AF, and CAD).

**Table 4:** Relative Risk of Postoperative Adverse Events Associated With CMR-LAEF <30%

	HR (95% CI)	*P* Value
Univariable analysis		
CMR-LAEF <30%	6.22 (3.10-12.46)	**<0.001**
Multivariable analysis		
Age	1.03 (1.00-1.07)	0.061
Women	2.55 (1.24-5.25)	**0.011**
History of atrial fibrillation	2.26 (1.06-4.81)	**0.034**
Coronary artery disease	3.33 (1.55-7.16)	**0.002**
CMR-LAEF <30%	3.78 (1.83-7.80)	**<0.001**

**Bold** indicates statistical significance (*P* < 0.05).

Abbreviations as in Table 2.

**Figure 3:**
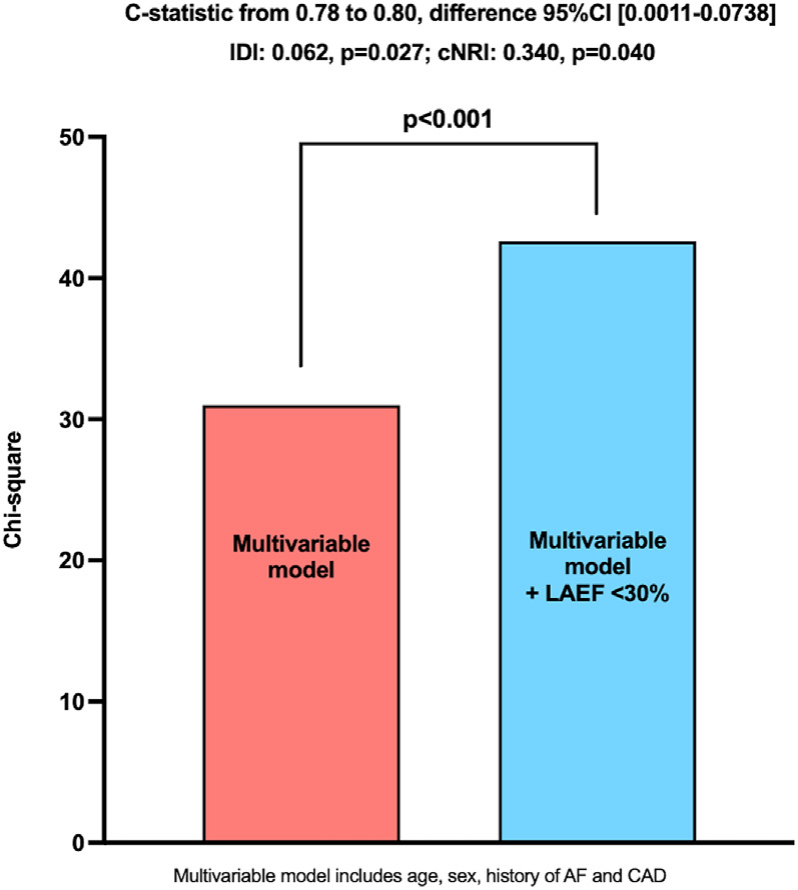
Incremental Prognostic Value of Cardiac Magnetic Resonance-Left Atrial Emptying Fraction <30% These bar plots display the incremental prognostic value of LAEF <30% over clinical covariates of prognostic importance in primary MR (age, sex, history of AF, CAD), as shown by better reclassification indices (IDI, cNRI), increase in chi-square, and C-statistic. AF = atrial fibrillation; CAD = coronary artery disease; cNRI = continuous net reclassification improvement; IDI = integrated discrimination improvement index; MR = mitral regurgitation; other abbreviations as in Figure 2.

The addition of CMR-LAEF <30% to the multivariable model resulted in a significant increase in the chi-square model (from 31 to 42.6, *P* < 0.001), an increase in C-statistic (from 0.78 to 0.80, C-statistic difference 95% CI: 0.0011-0.0738), and reclassification indices (integrative discrimination index: 0.062, *P* = 0.027; continuous net reclassification index: 0.340, *P* = 0.040) at 5 years (Figure 3).

When analysis was restricted to patients in sinus rhythm at the time of preoperative assessment (n = 266, 27 events), after adjusting for EuroSCORE II, those with CMR-LAEF <30% still displayed an increased risk of postoperative events compared with those with CMR-LAEF ≥30% (adjusted HR: 4.05 [95% CI: 1.66-9.88]; *P* < 0.001). When analysis was restricted to patients who did not undergo CABG during MV surgery (n = 256, 25 events), after adjusting for EuroSCORE II, the relationship between CMR-LAEF <30% and outcome remained (adjusted HR: 7.16 [95% CI: 2.87-17.89]; *P* < 0.001).

### Factors associated with CMR-LAEF

We assessed relations between CMR-LAEF and preoperative clinical and imaging characteristics (Supplemental Figure 1). Then, we performed a linear multivariable regression model including covariates that were significantly associated with CMR-LAEF on univariable analyses (Supplemental Table 5). Factors independently associated with CMR-LAEF were a history of AF, EuroSCORE II, CMR-RegFrac, and CMR-LVEF (all *P* < 0.019). Further incorporation of tricuspid regurgitation peak pressure gradient in this model when available (n = 238) yielded similar results.

After adjustment for history of AF, EuroSCORE II, CMR-RegFrac, and CMR-LVEF, CMR-LAEF <30% remained associated with increased risk of adverse events (adjusted HR: 4.64 [95% CI: 2.09-10.32]; *P* < 0.001) (Supplemental Table 6).

### Reproducibility

Inter- and intra-observer variabilities of CMR-LAEF were good, with an intraclass correlation coefficient of 0.90 (0.66-0.98) and 0.97 (0.79-0.99), respectively.

## Discussion

In this cohort of patients with significant primary MR, preoperatively assessed by CMR and echo, who underwent MV surgical repair, we found that reduced LAEF, right ventricular ejection fraction, and aortic forward indexed stroke volume were each associated with an increased risk of postoperative long-term clinical adverse outcomes. After multiple adjustments, we established an independent link between CMR-LAEF and postoperative adverse prognosis. For clinical practice, we identified a 30% CMR-LAEF threshold related to a 3.8-fold increased risk of adverse events, on top of clinical, echocardiographic, and other CMR preoperative characteristics, and with added prognostic value tested by multiple performance model metrics. Hence, these findings underscore the prognostic importance of LA function in primary MR and support that the preoperative assessment of CMR-LAEF could aid in tailoring clinical risk stratification of these patients.

### Prognostic value of CMR preoperative characteristics in primary MR

Previous CMR outcome studies in primary MR patients focused on clinical endpoints upstream of MV surgery.^9^^,^^10^ We aimed to go 1 step further by investigating the relationship between CMR preoperative characteristics and postoperative adverse events. After adjusting for age and sex, we found that decreased indexed aortic forward stroke volume, decreased CMR-RVEF, and decreased CMR-LAEF were linked to adverse outcome after MV surgery, consistent with echocardiographic studies.^7^^,^^17^^,^^18^ Patients experiencing adverse events in our study had lower CMR-RegVol and indexed LV end-diastolic volumes, which may appear counterintuitive. This finding can reasonably be attributed to the older age and higher proportion of women in patients who had postoperative adverse events, both characteristics associated with lower mitral regurgitant and ventricular volumes.^19^ After adjusting for age and sex, no significant association was found between indexed LV volumes and clinical adverse outcomes. Unlike previous studies, we did not observe any relationship between MR quantitative parameters (CMR-RegVol and CMR-RegFrac) and adverse events.^9^^,^^10^ This divergence is likely explained by the focus of these studies on asymptomatic patients with moderate or severe primary MR, analyzing outcomes upstream of MV surgery, while we focused on patients with significant primary MR who were scheduled for MV surgery at the time of preoperative assessment. Consequently, our findings, aligning with recent literature, indicate that postoperative outcomes are predominantly influenced by the hemodynamics and structural damage caused by MR rather than the valvular regurgitant load addressed by MV surgery.^12^^,^^20^

### Interplay between left atrial function, MR severity, and outcome

We identified a link between impaired preoperative LA function and postoperative adverse outcome in primary MR. This builds upon earlier research highlighting the prognostic importance of LA reservoir function assessed by speckle-tracking echocardiography in these patients.^8^^,^^21^ Butts et al elegantly underscored the interplay between extensive LA fibrosis, increased LA size, and decreased CMR-LAEF in primary MR and further linked LA function with LV systolic and diastolic myocardial remodeling and function through CMR tissue tagging.^22^^,^^23^ Inciardi et al explored the intricate relationship between decreasing LA function, assessed by echocardiography, and increasing MR-EROA in MR.^24^ This investigation highlighted the interplay between MR severity, LA function, and pulmonary pressures, with the latter, as demonstrated by Bakkestrøm et al being a major driver of symptoms in MR.^25^

### Prognostic value of CMR-LAEF in primary MR

Our hypothesis that reduced CMR-LAEF would correlate with adverse outcomes in primary MR had been reinforced by recent studies that underscored the relationship between CMR-LAEF and clinical events in ischemic cardiomyopathy, heart failure, or asymptomatic individuals without cardiovascular diseases selected from the MESA (Multi-Ethnic Study of Atherosclerosis) cohort.^26^, ^27^, ^28^ We identified that reduced CMR-LAEF was associated with an increased risk of postoperative adverse events in primary MR patients. Notably, patients experiencing adverse events, compared to those who did not, had similar CMR-indexed LAVmax but higher indexed LAVmin, resulting in lower CMR-LAEF values. Thus, our data suggest that the CMR assessment of LA function over size refines clinical risk stratification in primary MR, as previously reported in large-scale echocardiographic studies.^7^^,^^8^ Unlike CMR-RVEF or indexed aortic forward stroke volume, the relationship between CMR-LAEF and outcome persisted after adjusting for known clinical risk markers in primary MR. Additionally, we identified that increased EuroSCORE II, a history of AF, increased CMR-RegFrac, and decreased CMR-LVEF were independently associated with decreased CMR-LAEF. After adjusting for these characteristics, CMR-LAEF <30% remained associated with risk of postoperative adverse events, thereby reinforcing the clinical relevance of CMR-LAEF in the preoperative decision-making process of these patients.

### Clinical implications

We identified an optimal CMR-LAEF threshold of 30% using spline curves. This threshold allowed us to identify a subgroup of patients at significantly higher risk of postoperative adverse outcomes, even after successful MV repair surgery. These patients were older, more frequently women, and displayed features of more advanced valvular heart disease. Therefore, our results indicate that reduced preoperative CMR-LAEF should trigger close surveillance following MV surgery. Moreover, these findings, aligning with previous literature, support that MV surgery should be discussed before the onset of impaired LA function.

### Study limitations

The results of the present study should be interpreted considering its limitations. As patients were not enrolled consecutively, the potential for selection bias cannot be excluded. Retrospective follow-up data carries inherent limitations. We observed a strong relationship between reduced preoperative CMR-LAEF and postoperative adverse outcomes in significant primary MR, but with the design of the present study, we cannot demonstrate cause-effect relationship. Statistical inference was based on a moderate number of events (n = 36 [13%]). This finding is not unexpected, as the risk of clinical postoperative adverse events is low for most primary MR patients in contemporary practice.^29^ Herein, reduced CMR-LAEF was associated with an increased risk of adverse events, with a strong effect size that remained significant after multiple adjustments, assessed using 2 Cox multivariable models: the first based on a parsimonious selection of characteristics associated with adverse prognosis, and the second based on factors associated with CMR-LAEF. The added prognostic value of CMR-LAEF was confirmed through various performance model metrics. Also, to address the potential risk of model overfitting, the robustness of the Cox multivariable models was confirmed using bootstrapping and Poisson regression analysis.^30^ However, bootstrapping does not address issues related to sparse representation within covariate combinations, which may limit the stability and precision of estimates within strata including none or very few observations (see Supplemental Table 4). We deliberately focused on a homogeneous group of patients with chronic primary MR due to prolapse or flail undergoing MV repair surgery, who usually have fewer comorbidities than those with rheumatic or secondary MR. Further studies are needed to evaluate the clinical relevance of CMR-LAEF in other MR etiologies or in patients undergoing MV replacement. LA function was assessed by CMR-LAEF using the biplane area-length method. LA strain measurements allow a comprehensive understanding of the LA physiology by studying separately its different phases (reservoir, conduit, and pump) but were not available in our study. Nevertheless, LA strain measurements require dedicated CMR postprocessing software and are likely to be vendor-dependent, while CMR-LAEF benefits from being measurable in routine CMR studies.^31^ Alfuhied et al reported that CMR-LAEF had better reproducibility compared to LA strain.^32^ Another study showed comparable associations of CMR-LAEF and LA strain with diastolic dysfunction and outcome.^33^ Echo-derived LAEF and LA strain were not prospectively assessed in the vast majority of the study sample. Therefore, a head-to-head comparison between the prognostic value of CMR-LAEF and that of Echo-LAEF or LA strain could not be performed and would merit further investigation. Data regarding intraoperative hemodynamics were not available. While patients with early postoperative MR ≥ moderate were excluded, MR recurrence during long-term follow-up was not consistently documented, which may have introduced residual confounding. Myocardial T1 mapping and extracellular volume quantification emerged as promising risk markers in primary MR but were not available for much of this study sample. Lastly, we focused on postoperative clinical outcomes, preventing our ability to assess the relationship between CMR-LAEF and outcome upstream of the intervention. We believe our findings would pave the way for further studies to validate the proposed CMR-LAEF threshold of <30%, externally confirm its clinical relevance, and, crucially, investigate the role of CMR-LAEF in the decision-making process for primary MR patients upstream of MV surgery, beyond current recommended triggers for surgical intervention.

## Conclusions

In patients with significant primary MR due to prolapse or flail undergoing MV surgery, reduced preoperative CMR-LAEF was markedly associated with an increased risk of clinical postoperative long-term adverse outcome, independent of clinical risk factors, echocardiographic, and other CMR preoperative characteristics, and with added prognostic value. For clinical practice, CMR-LAEF <30% identifies a subgroup of patients at higher risk of adverse events who require close surveillance following MV surgery. Overall, our findings support the clinical relevance of evaluating LA function alongside size to tailor risk stratification for patients with primary MR.

Perspectives **COMPETENCY IN MEDICAL KNOWLEDGE:** Long-standing MR affects cardiac remodeling and hemodynamics, both of which influence postoperative prognosis even after successful MV surgery. We assessed the relationship between CMR preoperative characteristics and postoperative clinical outcomes in primary MR. On multivariable analysis, reduced CMR-LAEF was independently associated with risk of postoperative adverse outcomes, with its prognostic value validated by multiple performance metrics. We identified an optimal CMR-LAEF threshold of 30%, which delineates a subgroup of patients at higher risk of adverse events requiring close surveillance following MV surgery. These findings highlight the importance of evaluating LA function in addition to size to refine risk stratification in primary MR.

**TRANSLATIONAL OUTLOOK:** Further studies are warranted to validate the CMR-LAEF threshold of <30%, externally confirm its clinical relevance, and investigate the clinical utility of CMR-LAEF in the decision-making process for primary MR patients upstream of MV surgery, beyond current recommended triggers for surgical intervention.

## Funding support and author disclosures

This study was funded by FRSM CDR-35275164 of the Belgian Fondation de la Recherche Scientifique. The authors have reported that they have no relationships relevant to the contents of this paper to disclose.

## References

[1] Lazam S., Vanoverschelde J.L., Tribouilloy C. (2017). Twenty-year outcome after mitral repair versus replacement for severe degenerative mitral regurgitation: analysis of a large, prospective, multicenter, international registry. Circulation.

[2] Vahanian A., Beyersdorf F., Praz F. (2022). 2021 ESC/EACTS Guidelines for the management of valvular heart disease. Eur Heart J.

[3] Otto C.M., Nishimura R.A., Bonow R.O. (2021). 2020 ACC/AHA guideline for the management of patients with valvular heart disease: executive summary: a report of the American College of Cardiology/American Heart Association Joint Committee on clinical practice guidelines. J Am Coll Cardiol.

[4] Bernard J., Altes A., Dupuis M. (2022). Cardiac damage staging classification in asymptomatic moderate or severe primary mitral regurgitation. Structural Heart.

[5] van Wijngaarden A.L., Mantegazza V., Hiemstra Y.L. (2022). Prognostic impact of extra-mitral valve cardiac involvement in patients with primary mitral regurgitation. JACC Cardiovasc Imaging.

[6] Essayagh B., Antoine C., Benfari G. (2019). Prognostic implications of left atrial enlargement in degenerative mitral regurgitation. J Am Coll Cardiol.

[7] Essayagh B., Benfari G., Antoine C. (2022). Incremental prognosis by left atrial functional assessment: the left atrial coupling index in patients with floppy mitral valves. J Am Heart Assoc.

[8] Stassen J., van Wijngaarden A.L., Butcher S.C. (2022). Prognostic value of left atrial reservoir function in patients with severe primary mitral regurgitation undergoing mitral valve repair. Eur Heart J Cardiovasc Imaging.

[9] Myerson S.G., d’Arcy J., Christiansen J.P. (2016). Determination of clinical outcome in mitral regurgitation with cardiovascular magnetic resonance quantification. Circulation.

[10] Penicka M., Vecera J., Mirica D.C., Kotrc M., Kockova R., Van Camp G. (2018). Prognostic implications of magnetic resonance-derived quantification in asymptomatic patients with organic mitral regurgitation: comparison with Doppler echocardiography-derived integrative approach. Circulation.

[11] Uretsky S., Animashaun I.B., Sakul S. (2022). American Society of Echocardiography algorithm for degenerative mitral regurgitation: comparison with CMR. JACC Cardiovasc Imaging.

[12] Uretsky S., Biederman R.W.W., Han Y. (2023). Symptoms, outcomes, and regurgitant severity in guideline-directed mitral valve surgery: a multicenter prospective study. JACC Cardiovasc Imaging.

[13] Nashef S.A., Roques F., Sharples L.D. (2012). EuroSCORE II. Eur J Cardiothorac Surg.

[14] Lang R.M., Badano L.P., Mor-Avi V. (2015). Recommendations for cardiac chamber quantification by echocardiography in adults: an update from the American Society of Echocardiography and the European Association of Cardiovascular Imaging. J Am Soc Echocardiogr.

[15] Schulz-Menger J., Bluemke D.A., Bremerich J. (2020). Standardized image interpretation and post-processing in cardiovascular magnetic resonance - 2020 update : society for cardiovascular magnetic resonance (SCMR): board of trustees task force on standardized post-processing. J Cardiovasc Magn Reson.

[16] Petersen S.E., Aung N., Sanghvi M.M. (2017). Reference ranges for cardiac structure and function using cardiovascular magnetic resonance (CMR) in Caucasians from the UK Biobank population cohort. J Cardiovasc Magn Reson.

[17] Granot Y., Gefen S., Karlsberg D. (2024). Prognostic value of echocardiographic-derived stroke volume in severe primary mitral regurgitation. Eur Heart J Cardiovasc Imaging.

[18] Bohbot Y., Essayagh B., Benfari G. (2024). Prognostic implications of right ventricular dysfunction in severe degenerative mitral regurgitation. J Am Heart Assoc.

[19] Altes A., Levy F., Hanet V. (2024). Impact of sex on severity assessment and cardiac remodeling in primary mitral regurgitation. JACC Adv.

[20] Butcher S.C., Essayagh B., Steyerberg E.W. (2023). Factors influencing post-surgical survival in degenerative mitral regurgitation. Eur Heart J.

[21] Mandoli G.E., Pastore M.C., Benfari G. (2021). Left atrial strain as a pre-operative prognostic marker for patients with severe mitral regurgitation. Int J Cardiol.

[22] Butts B., Ahmed M.I., Bajaj N.S. (2020). Reduced left atrial emptying fraction and chymase activation in pathophysiology of primary mitral regurgitation. JACC Basic Transl Sci.

[23] Ahmed M.I., Andrikopoulou E., Zheng J. (2022). Interstitial collagen loss, myocardial remodeling, and function in primary mitral regurgitation. JACC Basic Transl Sci.

[24] Inciardi R.M., Rossi A., Bergamini C. (2020). Mitral regurgitation, left atrial structural and functional remodelling and the effect on pulmonary haemodynamics. Eur J Heart Fail.

[25] Bakkestrøm R., Banke A., Christensen N.L. (2018). Hemodynamic characteristics in significant symptomatic and asymptomatic primary mitral valve regurgitation at rest and during exercise. Circ Cardiovasc Imaging.

[26] Anthony C.M., Wang T.K.M., Salam D. (2024). Impact of cardiac magnetic resonance left atrial ejection fraction in advanced ischemic cardiomyopathy. JACC Adv.

[27] Habibi M., Samiei S., Ambale Venkatesh B. (2016). Cardiac magnetic resonance-measured left atrial volume and function and incident atrial fibrillation: results from MESA (Multi-Ethnic study of Atherosclerosis). Circ Cardiovasc Imaging.

[28] Pellicori P., Zhang J., Lukaschuk E. (2015). Left atrial function measured by cardiac magnetic resonance imaging in patients with heart failure: clinical associations and prognostic value. Eur Heart J.

[29] Khan F.W., Greason K.L., King K.S. (2024). Population-based analysis of late outcomes of mitral valve repair for degenerative mitral valve regurgitation. JACC Adv.

[30] Figliozzi S., Georgiopoulos G., Lopes P.M. (2023). Myocardial fibrosis at cardiac MRI helps predict adverse clinical outcome in patients with mitral valve prolapse. Radiology.

[31] Pathan F., Zainal Abidin H.A., Vo Q.H. (2021). Left atrial strain: a multi-modality, multi-vendor comparison study. Eur Heart J Cardiovasc Imaging.

[32] Alfuhied A., Marrow B.A., Elfawal S. (2021). Reproducibility of left atrial function using cardiac magnetic resonance imaging. Eur Radiol.

[33] Nguyen J., Weber J., Hsu B., Mulyala R.R., Wang L., Cao J.J. (2021). Comparing left atrial indices by CMR in association with left ventricular diastolic dysfunction and adverse clinical outcomes. Sci Rep.

