# Supplementary material for: Prognostic Implications of Cardiac Magnetic Resonance Imaging Characteristics in Primary Mitral Regurgitation
Source: JACC Adv. 2025 Jun 25;4(6):101838. doi: 10.1016/j.jacadv.2025.101838 (PMC12287956; doi:10.1016/j.jacadv.2025.101838)
Supplement: Supplemental data [file mmc1.docx]

**Supplemental Appendix**

**Cardiac magnetic resonance imaging**

CMR studies were performed on clinical scanners (Brussels: 1.5 T Intera CV/ 3T Ingenia CV, Philips Medical System, Best, the Netherlands, or 3-T Signa, General Electrics, USA; Lille: 3-T General Electrics or 1.5-T Altea, Siemens Medical Solutions, Erlangen, Germany) and analyzed locally using either Segment v3.0 (http://segment.heiberg.se), cvi42^©^, Circle Cardiovascular Imaging, Calgary, AB, Canada or Syngovia^©^, Siemens Medical Systems) software. Ventricular chamber assessment was performed in accordance with guidelines. LV and right ventricular (RV) EDV, ESV and EF were assessed from consecutive short-axis cine balanced steady state free precession pulse (bSSFP) images covering the entire LV from the mitral plane to the apex and analyzed by experienced operators blinded to the echocardiographic data of the patient. LV mass was obtained at end-diastole with trabeculations and papillary muscles included in volumes, assuming myocardial density of 1.05 g/cm^2^. The LV mass, LV and RV volumes were indexed to BSA (calculated using the Mosteller formula). The LV total stroke volume (LVTSV) was obtained by subtracting the LVESV from the LVEDV. Aortic stroke volume was derived from quantitative through-plane phase-contrast measurement performed at the level of the sino-tubular junction perpendicular to the aorta. Mitral regurgitant volume (CMR-RegVol) was calculated by subtracting the aortic stroke volume from the total LVTSV (obtained from cine-volumetric CMR acquisition). Mitral regurgitant fraction (CMR-RegFrac) was defined as CMR-RegVol divided by the LVTSV, expressed as a percentage. Late gadolinium enhancement (LGE) imaging was performed in the majority of patients (n=236) 10 to 15 minutes after intravenous administration of gadolinium contrast agent using inversion-recovery pulse sequences. The decision to perform LGE imaging during CMR acquisition was left to the discretion of the practitioner. Consequently, since the primary purpose of CMR in these patients was typically MR quantification, LGE imaging was not systematically conducted for every patient. LA volumes were retrospectively contoured by a single investigator (A.A), blinded to clinical and outcome data, from the 2‐chamber and 4‐chamber cine images in the ventricular end‐systolic phase immediately before MV opening (LA maximal volume [CMR-LAVmax]) and the end‐diastolic phase right after MV closure (LA minimal volume [CMR-LAVmin]) using the biplane-area formula (**Figure 1**). For LA volume assessment, the pulmonary veins and LA appendage were excluded. LA emptying fraction (LAEF) was calculated as follows: (LAVmax-LAVmin)/LAVmax, expressed as a percentage. To assess intraobserver reproducibility, the main investigator (A.A.) repeated the analysis using a randomly chosen subset of 10 patients at ≥14 days after the first analysis. To assess interobserver reproducibility, those 10 randomly chosen examinations were analyzed by 2 investigators (A.A. and S.M.).

**Statistical analysis**

Data were analyzed with R version 4.1.1 (R Foundation for Statistical Computing, Vienna, Austria) and GraphPad Prism (GraphPad Software, La Jolla, California). Quantitative data are reported as median [25th–75th percentile], while qualitative data are presented as absolute numbers and percentages. Time-to-event analyses were conducted using Cox proportional hazards models. The multivariable Cox model was built using stepwise backward selection, starting from a full model including all candidate covariates (those associated with outcome in univariable analyses: age, female sex, history of AF, eGFR, NYHA class ≥ 3, EuroSCORE II, CAD, CMR-ind aortic forward stroke volume, CMR-RVEF and CMR-LAEF). Variable selection was based on the Akaike Information Criterion (AIC), using the stepAIC() function from the MASS R package (version 7.3-58.1). This method iteratively removed covariates that did not contribute to model performance, aiming to achieve an optimal balance between goodness-of-fit and parsimony. After backward selection, covariates retained in the final Cox multivariable model were: age, female sex, history of AF, CAD and CMR-LAEF. The proportional hazards assumption was assessed using Schoenfeld residuals and log-log plots. Martingale residuals were used to detect nonlinearity in continuous variables. Multicollinearity was tested through the Variance Inflation Factor (VIF < 5).

Then, patients were stratified by CMR-LAEF ≥ or < 30 % according to the threshold identified with the use of spline curves. Associations between the two groups and baseline categorical variables were examined using either Pearson chi-square statistic or Fisher’s exact test. Individual differences for continuous variables were compared using Mann–Whitney U tests. Intraobserver and interobserver reproducibility of CMR-LAEF were evaluated using intraclass correlation coefficient (ICC, single rater/measurement, absolute-agreement, two-way fixed-effects model).

Survival until primary endpoint was evaluated from the day of MV surgery. Median follow-up time was obtained by the reverse Kaplan-Meier method. Event free survival was estimated according to the Kaplan-Meier method and compared using two-sided log-rank tests and univariable and multivariable Cox proportional hazards models. Patients who experienced more than one event were censored at the time of the first event. Because of the moderate number of clinical events, and to account for the risk of model overfitting, the results of the main multivariable Cox regression model were confirmed using bootstrapping with 1000 replicates and Poisson regression with a robust sandwich estimator to account for variability in the data. For the full assessment of the incremental benefit of LAEF < 30%, we compared the potential additive prognostic value of LAEF < 30% to improve the prediction of adverse events over clinical covariates using multiple discrimination measures including likelihood ratio and the additional increase of χ2 of the combined model over the baseline model, net reclassification improvement (NRI), the integrative discrimination improvement index (IDI), and by estimating the Harrell’s C-statistic for models with and without LAEF < 30%. To enable comparison between C statistics, a total of 999 bootstrap samples of the patients in our study sample were generated using the library (boot) in R, and the difference in Harrell C statistics between the models with and without LAEF < 30% was computed for each of the 999 samples. We hence obtained the 95% bootstrap CIs of the 999 estimates, for which the lower and upper bounds were the 2.5^th^ and 97.5^th^ percentiles of the resampling distribution, respectively. NRI and IDI were computed at 5 years using the R package survIDINRI (version 1.1-2). Factors associated with CMR-LAEF were analyzed using Pearson’s coefficient correlations and linear regression models. All analyses considered a two-tailed p value of < 0.05 as statistically significant.

**Supplemental Figure 1. Associations between CMR-LAEF and preoperative clinical and imaging characteristics**

This barplot displays the Pearson’s correlation coefficients of preoperative clinical and imaging covariates with CMR-LAEF. Positive correlations are displayed to the right, while negative correlations are displayed to the left. LA volumes were not included in this analysis because they are part of CMR-LAEF calculation.

**
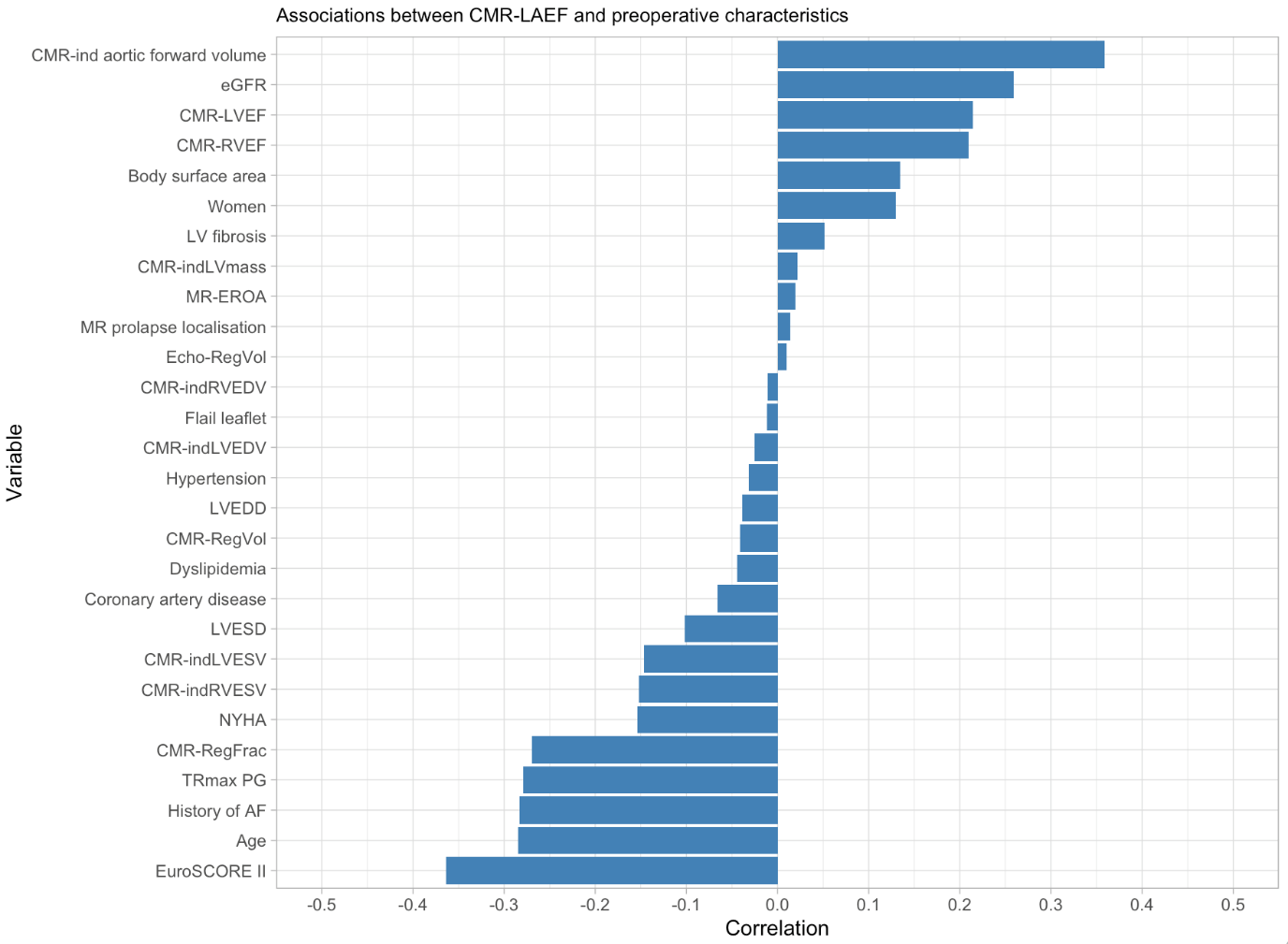
**

**Supplemental Table 1: Demographic, clinical and surgical characteristics according to LA emptying fraction < or ≥ 30%**

| **Variable** | **All**  **N = 284** | **CMR-LAEF ≥ 30%**  **n = 250** | **CMR-LAEF < 30%**  **n = 34** | **Overall**  **p-value** |
| --- | --- | --- | --- | --- |
| **Clinical characteristics** |  |  |  |  |
| Age (years) | 61 [51;69] | 59 [51;67] | 70 [63;75] | **<0.001** |
| Women, n (%) | 69 (24%) | 55 (22%) | 14 (41%) | **0.026** |
| Body surface area (m^2^) | 1.93 [1.79;2.05] | 1.94 [1.81;2.05] | 1.83 [1.63;2.01] | **0.011** |
| Body mass index (kg/m²) | 24.7 [22.7;26.9] | 24.7 [22.7;26.9] | 24.5 [22.6;26.4] | 0.331 |
| NYHA functional class, n (%) |  |  |  | 0.076 |
| - I | 118 (42%) | 110 (44%) | 8 (23.5%) |  |
| - II | 108 (38%) | 91 (36%) | 17 (50%) |  |
| - III/IV | 58 (20%) | 49 (20%) | 9 (26.5%) |  |
| Hypertension, n (%) | 109 (38%) | 94 (38%) | 15 (44%) | 0.586 |
| Diabetes mellitus, n (%) | 7 (2%) | 7 (3%) | 0 (0%) | 0.605 |
| Dyslipidemia, n (%) | 84 (30%) | 74 (30%) | 10 (29%) | 1.000 |
| Coronary artery disease, n (%) | 40 (14%) | 33 (13%) | 7 (21%) | 0.297 |
| History of atrial fibrillation, n (%) | 47 (16.5%) | 32 (13%) | 15 (44%) | **<0.001** |
| eGFR (ml/min/1.73 m^2^) | 88 [66;110] | 90 [70;112] | 62 [53;85] | **<0.001** |
| **Surgical characteristics** |  |  |  |  |
| EuroSCORE II (%) | 0.99 [0.68;1.70] | 0.96 [0.67;1.48] | 2.01 [1.11;3.29] | **<0.001** |
| Surgical approach, n (%) : |  |  |  | 0.211 |
| - Robotic-assisted surgery | 69 (24%) | 66 (26%) | 3 (9%) |  |
| - Median sternotomy | 193 (68%) | 164 (66%) | 29 (85%) |  |
| -Video-assisted minimally invasive surgery | 22 (8%) | 20 (8%) | 2 (6%) |  |
| Associated CABG, n (%) | 28 (10%) | 21 (8%) | 7 (21%) | **0.026** |
| Associated tricuspid annuloplasty, n (%) | 91 (32%) | 80 (32%) | 11 (32%) | 1.000 |

Legend: CABG = coronary artery bypass graft; eGFR = estimated glomerular filtration rate; MV = mitral valve; NYHA = New York Heart Association

**Supplemental Table 2: Echocardiographic and CMR characteristics according to LA emptying fraction < or ≥ 30%**

| **Variable** | **All**  **N = 284** | **CMR-LAEF ≥ 30%**  **n = 250** | **CMR-LAEF < 30%**  **n = 34** | **Overall**  **p-value** |
| --- | --- | --- | --- | --- |
| **Echocardiographic characteristics** |  |  |  |  |
| LVEDD (mm) | 58 [55;62] | 58 [54;62] | 58 [55;62] | 0.813 |
| LVESD (mm) | 35 [31;40] | 35 [31;40] | 38 [33;41] | 0.175 |
| Echo-LVEF (%) | 64 [60;70] | 65 [60;69] | 64 [61;70] | 0.911 |
| Echo-indLAV (ml/m^2^) | 54 [43;71] | 53 [42;68] | 71 [52;84] | **0.001** |
| TRmax PG (mmHg) (n=238) | 26 [21;35] | 25 [21;33] | 35 [27.5;43.5] | **0.001** |
| MR-EROA (mm^2^) (n=269) | 51 [40;65] | 50 [40;64] | 51 [38;70] | 0.912 |
| Echo-RegVol (ml) (n=269) | 75 [62;91] | 75 [62;91] | 79 [60;88] | 0.723 |
| MR prolapse localisation, n (%) |  |  |  | 0.956 |
| - Anterior | 23 (8%) | 20 (8%) | 3 (9%) |  |
| - Posterior | 201 (71%) | 178 (71%) | 23 (68%) |  |
| - Bi-leaflet | 60 (21%) | 52 (21%) | 8 (23.5%) |  |
| Flail leaflet, n (%) | 122 (43%) | 107 (43%) | 15 (44%) | 1.000 |
| **CMR characteristics** |  |  |  |  |
| CMR-indLVEDV (ml/m^2^) | 116 [99;129] | 116 [101;129] | 116 [92.5;128] | 0.541 |
| CMR-indLVESV (ml/m^2^) | 42 [34;52] | 42 [34;52] | 43 [35;56] | 0.431 |
| CMR-indLV mass (g/m^2^) | 74.5 [66;82.5] | 74.5 [67;82] | 74 [62;82] | 0.494 |
| CMR-LVEF (%) | 63 [58;67] | 64 [58;67] | 60 [54.5;64] | **0.028** |
| CMR-ind aortic forward volume (ml/m^2^) | 37 [30;42] | 38 [32;43] | 29 [24;36] | **<0.001** |
| CMR-RegVol (ml) | 62 [47;86] | 62 [46;84.5] | 61 [50;91] | 0.834 |
| CMR-RegFrac (%) | 49 [38;57] | 47 [37;56] | 56 [44.5;64] | **0.001** |
| CMR-indRVEDV (ml/m^2^) | 77 [66;90] | 78 [66;90] | 77 [64;89] | 0.828 |
| CMR-indRVESV (ml/m^2^) | 38 [30;47] | 37 [30;47] | 41 [30;51] | 0.384 |
| CMR-RVEF (%) | 51 [46;56] | 51 [47;56] | 47 [43;56] | 0.094 |
| LV fibrosis, n (%) (n=236) | 51 (22%) | 47 (22%) | 4 (15%) | 0.572 |
| CMR-ind end-systolic LA volume (ml/m^2^) | 76 [61;98] | 73.5 [59;92.5] | 106 [89.5;131] | **<0.001** |
| CMR-ind end-diastolic LA volume (ml/m^2^) | 42 [31;58] | 39 [29;53] | 76 [71;107] | **<0.001** |
| CMR-LAEF (%) | 44 [37;52] | 45 [40;53] | 21 [16;26] | **<0.001** |

Legend: EDD = end-diastolic diameter, EDV = end-diastolic volume; EF = ejection fraction; ESD = end-systolic diameter; ESV = end-systolic volume; ind = indexed to body surface area; LAV = left atrial volume; LAEF = left atrial emptying fraction; LV = left ventricle/ventricular; MR-EROA = mitral regurgitant effective regurgitant orifice area; RegFrac = mitral regurgitant fraction, RegVol = mitral regurgitation volume, RV = right ventricle/ventricular, TRmax PG = tricuspid regurgitation peak pressure gradient

**Supplemental Table 3: Relative risk of adverse events associated with CMR-LAEF < 30% assessed by Cox multivariable model after bootstrap re-sampling (1000 times) and Poisson regression model**

|  | **Cox multivariable model after bootstrap re-sampling (1000 times)** | **Poisson regression model** |
| --- | --- | --- |
|  | Adjusted HR (95% CI) | Adjusted IRR (95% CI) |
| **CMR-LAEF < 30%** | 4.36 (1.68 – 8.84) | 3.36 (1.67 – 6.72) |
| **Age** | 1.04 (1.00 – 1.08) | 1.03 (1.00 – 1.07) |
| **Women** | 2.85 (1.13 – 5.81) | 2.71 (1.33 – 5.52) |
| **History of atrial fibrillation** | 2.47 (1.03 – 5.01) | 2.15 (1.02 – 4.52) |
| **Coronary artery disease** | 4.03 (1.36 – 9.30) | 3.04 (1.35 – 6.82) |

Legend: HR = hazard ratio, IRR = incidence rate ratio, LAEF = left atrial emptying fraction

**Supplemental Table 4: Distribution of events and non-events across 2×2×2 covariate combinations (sex, history of atrial fibrillation, and coronary artery disease)**

| Sex | History of AF | CAD | Event | Non-event |
| --- | --- | --- | --- | --- |
| Women | No | No | 9 | 48 |
| Women | No | Yes | 1 | 2 |
| Women | Yes | No | 5 | 3 |
| Women | Yes | Yes | 1 | 0 |
| Men | No | No | 7 | 142 |
| Men | No | Yes | 6 | 22 |
| Men | Yes | No | 5 | 25 |
| Men | Yes | Yes | 2 | 6 |

Legend: AF = atrial fibrillation, CAD = coronary artery disease

**Supplemental Table 5: Clinical and imaging characteristics associated with CMR-LAEF**

|  | **Unadjusted** |  | **Adjusted** |  |
| --- | --- | --- | --- | --- |
| **Covariates** | **β coefficient (95% CI)** | **P value** | **β coefficient (95% CI)** | **P value** |
| Age | -0.27 (-0.37, -0.16) | **<0.001** | -0.12(-0.26, 0.02) | 0.102 |
| Women | 3.63 (0.40, 6.87) | **0.029** | -2.21 (-5.82, 1.41) | 0.233 |
| Body surface area | 8.09 (1.15, 15.04) | **0.023** | 4.70 (-4.50, 13.90) | 0.317 |
| History of AF | -9.13 (-12.74, -5.52) | **<0.001** | -6.55 (-9.98, -3.12) | **<0.001** |
| Hypertension | -0.77 (-3.65, 2.10) | 0.600 |  |  |
| Dyslipidemia | -1.15 (-4.22, 1.91) | 0.461 |  |  |
| Coronary artery disease | -2.27 (-6.29, 1.74) | 0.268 |  |  |
| EuroSCORE II | -3.85 (-5.00, -2.70) | **<0.001** | -1.66 (-3.02, -0.30) | **0.018** |
| eGFR | 0.10 (0.06, 0.15) | **<0.001** | -0.02 (-0.09, 0.05) | 0.524 |
| LVEDD | -0.08 (-0.32, 0.16) | 0.517 |  |  |
| LVESD | -0.19 (-0.42, 0.03) | 0.090 |  |  |
| MR-EROA | 0.01 (-0.06, 0.08) | 0.751 |  |  |
| Echo-RegVol | 0.00 (-0.05, 0.06) | 0.874 |  |  |
| TRmax PG (n=238) | -0.29 (-0.42, -0.16) | **<0.001** |  |  |
| MR prolapse localisation | 0.33 (-2.34, 2.99) | 0.810 |  |  |
| Flail leaflet | -0.28 (-3.10, 2.55) | 0.847 |  |  |
| CMR-indLVEDV | -0.01 (-0.07, 0.05) | 0.675 |  |  |
| CMR-indLVESV | -0.12 (-0.22, -0.03) | **0.014** |  |  |
| CMR-indLV mass | 0.02 (-0.08, 0.12) | 0.722 |  |  |
| CMR-LVEF | 0.35 (0.16, 0.53) | **<0.001** | 0.31 (0.11, 0.52) | **0.003** |
| CMR-ind aortic forward volume | 0.47 (0.33, 0.61) | **<0.001** | 0.17 (-0.06, 0.40) | 0.141 |
| CMR-RegVol | -0.02 (-0.07, 0.03) | 0.491 |  |  |
| CMR-RegFrac | -0.27 (-0.38, -0.16) | **<0.001** | -0.18 (-0.33, -0.03) | **0.019** |
| CMR-indRVEDV | -0.01 (-0.08, 0.07) | 0.856 |  |  |
| CMR-indRVESV | -0.13 (-0.23, -0.03) | **0.010** | -0.07 (-0.21, 0.07) | 0.338 |
| CMR-RVEF | 0.30 (0.14, 0.46) | **<0.001** | -0.05 (-0.28, 0.19) | 0.698 |
| LV fibrosis | 1.52 (-2.26, 5.29) | 0.432 |  |  |
| Intercept | - | - | 42.58 (15.84, 69.32) | **0.002** |

This table displays univariable and multivariable linear regression analysis of the associations between CMR-LAEF and clinical or imaging characteristics. CMR-LVEF and CMR-indLVESV were not both included in the multivariable model because of collinearity.

**Supplemental Table 6: Relative risk of adverse events associated with CMR-LAEF < 30% after adjusting for history of AF, Euroscore II, CMR-RegFrac and CMR-LVEF**

|  | **Cox multivariable model** | **Cox multivariable model after bootstrap re-sampling (1000 times)** | **Poisson regression model** |
| --- | --- | --- | --- |
|  |  | Adjusted HR (95% CI) | Adjusted IRR (95% CI) |
| **CMR-LAEF < 30%** | 4.64 (2.09 - 10.32) | 5.44 (1.86 – 11.21) | 3.96 (1.81 – 8.65) |
| **History of AF** | 1.21 (0.98 - 1.49) | 2.01 (0.74 – 4.30) | 1.76 (0.81 – 3.82) |
| **EuroSCORE II** | 1.78 (0.84 - 3.79) | 1.26 (1.01 – 1.69) | 1.23 (1.04 – 1.46) |
| **CMR-RegFrac** | 1.00 (0.97 - 1.02) | 0.99 (0.96 – 1.02) | 0.99 (0.97 – 1.02) |
| **CMR-LVEF** | 1.04 (1 - 1.09) | 1.05 (0.98 – 1.12) | 1.04 (0.98 – 1.11) |

Legend: HR = hazard ratio, IRR = incidence rate ratio, LAEF = left atrial emptying fraction
